# Impact of the Mobile Game FightHPV on Cervical Cancer Screening Attendance: Retrospective Cohort Study

**DOI:** 10.2196/36197

**Published:** 2022-12-13

**Authors:** Madleen Orumaa, Suzanne Campbell, Nathalie C Støer, Philip E Castle, Sagar Sen, Ameli Tropé, Adebola Adedimeji, Mari Nygård

**Affiliations:** 1 Department of Research Cancer Registry of Norway Oslo Norway; 2 Department of Epidemiology and Biostatistics Institute for Health Development Tallinn Estonia; 3 Norwegian Research Centre for Women’s Health Women’s Clinic Oslo University Hospital Oslo Norway; 4 Divisions of Cancer Prevention and Cancer Epidemiology and Genetics National Cancer Institute Rockville, MD United States; 5 SINTEF Oslo Norway; 6 Section for Cervical Cancer Screening Cancer Registry of Norway Oslo Norway; 7 Department of Epidemiology and Population Health Albert Einstein College of Medicine New York, NY United States

**Keywords:** mobile app, gamification, empowering, health literacy, cervical cancer screening, cancer prevention

## Abstract

**Background:**

The wide availability of mobile phones has made it easy to disseminate health-related information and make it accessible. With gamification, mobile apps can nudge people to make informed health choices, including attending cervical cancer screening.

**Objective:**

This matched retrospective cohort study examined the association between exposure to the FightHPV mobile app gamified educational content and having a cervical exam in the following year.

**Methods:**

Women aged 20 to 69 years who signed an electronic consent form after downloading the FightHPV app in 2017 (intervention group) were matched 1:6 with women of the same age and with the same screening history (reference group) in 2015. To estimate the impact of exposure to the FightHPV app, we estimated cumulative incidence and hazard ratios (HRs) with 95% CIs. We used data from the Norwegian Cervical Cancer Screening Program database and Statistics Norway to determine screening participation and outcomes, respectively.

**Results:**

We matched 3860 women in the control group to 658 women in the intervention group; 6 months after enrollment, 29.6% (195/658) of the women in the intervention group and 15.21% (587/3860) of those in the reference group underwent a cervical exam (*P*<.01). Women exposed to the FightHPV app were 2 times more likely to attend screening (adjusted HR 2.3, 95% CI 2.0-2.7), during which they were 13 times more likely to be diagnosed with high-grade abnormality (adjusted HR 12.7, 95% CI 5.0-32.5) than the women in the reference group.

**Conclusions:**

Exposure to the FightHPV app significantly increased cervical cancer screening attendance across the various analyses and improved detection of women with high risk for cervical cancer. For the first time, we demonstrated the effectiveness of gamification combined with mobile technology in cancer prevention by empowering women to make active health-related decisions. Gamification can significantly improve the understanding of complicated scientific concepts behind interventions and increase the acceptance of proposed cancer control measures.

## Introduction

### Background

In 2019, the World Health Organization released the first evidence-based guideline on scaling up the use of digital interventions to reduce health inequities [1] and introduce a new era in health care [2]. Indeed, not only is successful dissemination of health-related information possible through the wide availability of mobile phones and social media, but these platforms also allow novel intervention designs for difficult-to-reach segments of the population, such as lower socioeconomical groups, youth, and older adults [3,4]. Theories suggest that health-related behavior is multidimensional and influenced by various factors, including information literacy [5-7]. Gamification is an innovative approach that motivates people to acquire new knowledge in a fun and engaging way [8], and it has proven to be a successful knowledge translation tool [9]. In eHealth, gamification has been widely used in various lifestyle mobile apps [9,10]; however, to our knowledge, its effectiveness has not yet been studied in a cancer prevention setting.

Cervical cancer screening aims to detect and treat cervical precancers, thereby reducing the cancer burden [11,12]. Screening is available in most high-income countries [13], where millions of women are invited to undergo free-of-charge or low-cost screening procedures every 3 to 5 years [11]. The most efficient, and often the most effective, cervical cancer screening is organized by public health programs, which promote equal access and ensure the quality of procedures related to screening exams and follow-up exams, diagnostic procedures for women with an abnormal screening exam result, and treatment of those with precancerous abnormalities or frank cancer.

Despite the general availability of cervical cancer screening in high-income settings, a subgroup of women is commonly observed not participating in these programs [14], leaving them at higher risk for the presence of precancerous abnormalities that may progress to cancer. A significant proportion, often >50%, of the cervical cancers diagnosed in these high-income settings are in unscreened and underscreened women [15]. Conversely, some women attend screening too frequently; that is, they attend at a shorter interval than is recommended. In a screening program context, frequent screening results in more harm, including detection and treatment of self-limiting conditions with a negative impact on women’s mental and physical health, and increased health care costs [16,17]. Both under- and overscreening are related to the lack of understanding of health-related information [18].

### Objectives

To improve the understanding of the importance of screening, we developed an innovative game-based mobile phone learning tool named FightHPV to educate its users about cervical cancer; its main causal agent, human papillomavirus (HPV); and its prevention. The FightHPV app has been documented to improve knowledge about screening, causes of cancer, and HPV [19]. A detailed description of the FightHPV app content, gameplay mechanics, and strategies used to engage the player can be found in Multimedia Appendix 1 (refer to Description of the FightHPV App [19-21]). However, it is still unclear whether exposure to the FightHPV app increases readiness to attend the cervical screening. To answer this question, we conducted a matched retrospective cohort study to examine the impact of being exposed to the FightHPV app gamified educational content and having a cervical exam in the following 12 months.

## Methods

### Overview

Our study took advantage of high-quality data from the Norwegian Cervical Cancer Screening Program (NCCSP) and Statistics Norway, both of which are nationwide registries with excellent completeness records with regard to collecting individual-level data over a long period of time. The study was restricted to Norway and to those who identified themselves through a nationally adapted, secure electronic BankID (a personal and simple electronic ID for secure identification used widely in Norway). The linkage between the study participants and the national registries ensured accurate information on outcomes and adjustment variables, such as education, employment status, income, and country of birth. These confounders have a documented effect on screening attendance in Norway [22] and might be associated with the use of health apps [23]. A more detailed description of the NCCSP and the linkage process is available in Multimedia Appendix 1 (refer to Description of the Norwegian Cervical Cancer Screening Program and the Linkage Process [24-27] and Figure S1).

### Study Design

#### Electronic Consent and Enrollment of the Intervention Group

**Figure 1:**
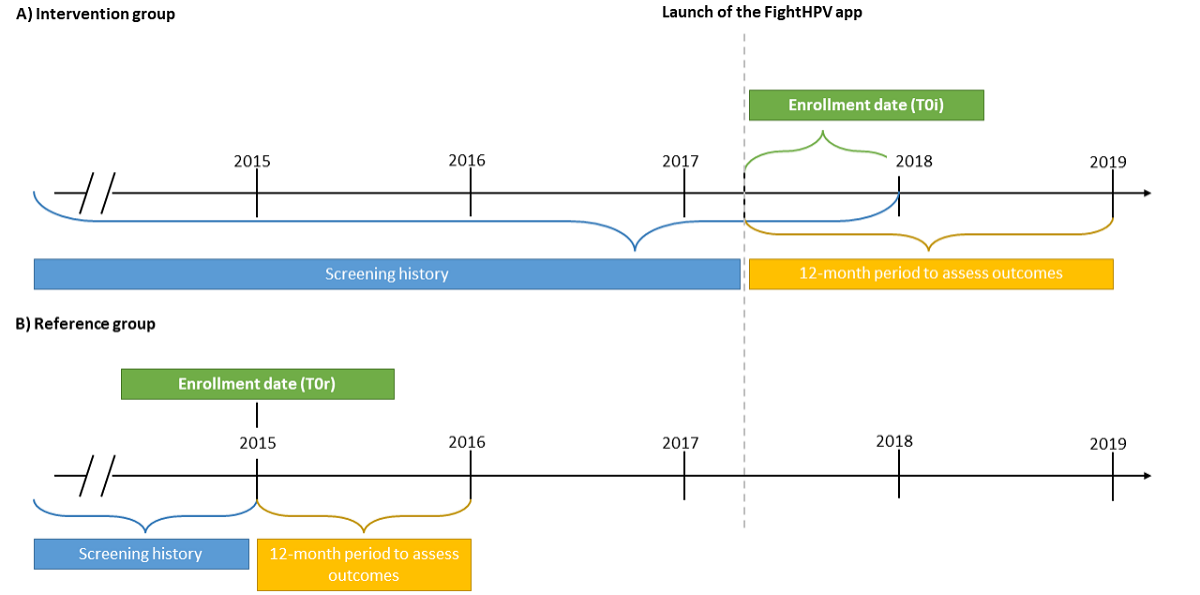
Relevant time points for enrollment, screening history, and assessment periods for both intervention and reference groups.

**Figure 2:**
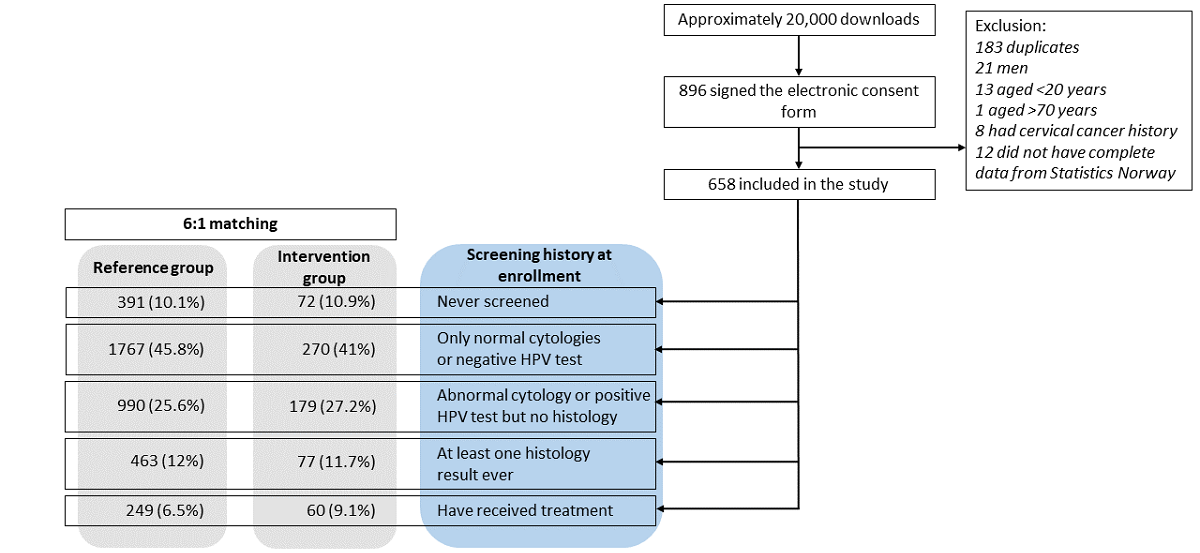
Selection of the intervention group and distribution of the screening history subgroups at enrollment for both the intervention (n=658) and reference (n=3860) groups. HPV: human papillomavirus.

Every player who downloaded the app from the Apple App Store or Google Play Store and was residing in Norway had an opportunity to participate in the research project. In total, 896 players provided electronic consent by identifying themselves via BankID and were enrolled to the intervention group. The time at which a signature was provided through the electronic consent was defined as the start of the follow-up (T0i). The enrollment period was between March 1, 2017, and December 31, 2017. Among these 896 players, after excluding 238 (26.6%) for various reasons, we identified 658 (73.4%) women aged 20 to 69 years who were subsequently included in the study (Figures 1 and 2). By using individual screening data from the NCCSP, we categorized the intervention group by screening history at the enrollment date (T0i) into five mutually exclusive subgroups (Figure 2): (1) never screened, (2) only normal cytology results or negative HPV test, (3) abnormal cytology or positive HPV test but no histology ever, (4) at least one histology result, or (5) have ever received treatment for cervical abnormalities.

#### Selection of the Reference Group

To avoid having been exposed to the FightHPV app launched in 2017, we defined January 1, 2015, as the enrollment date (T0r) for the reference group (Figure 1). Similar to the intervention group, we used NCCSP individual screening data and categorized all eligible women for the reference group into the previously described 5 mutually exclusive screening history subgroups on January 1, 2015 (enrollment date [T0r]). Women in the intervention group were matched 1:6 with women in the reference group by age at the enrollment date (T0) and screening history subgroup.

In total, 4818 participants were enrolled to the study and linked with Statistics Norway to obtain information on known confounders such as marital status, education, employment status, income, and country of birth. We used complete case analyses; therefore, participants with missing information regarding education (152/4818, 3.15%), country of birth (15/4818, 0.31%), employment status (46/4818, 0.95%), marital status (43/4818, 0.89%), or income (44/4818, 0.91%) were excluded. Thus, of the 4818 participants, after excluding 300 (6.23%), 658 (13.66%) were left in the intervention group and 3860 (80.12%) in the reference group for the analyses. After exclusions, the mean matching ratio was 1 case: 5.9 controls; 553 cases were matched 1:6, whereas 8 cases had <3 matches in the reference group (Figure 2).

### Statistics

The impact of exposure to the FightHPV app on having a cervical exam and on the cervical exam outcome was assessed during the 1-year period after the enrollment date (T0i and T0r; Figure 1). We defined having a cervical exam as positive if at least one cervical exam result (cytology, HPV test, or histology) was registered during the 1-year period after the enrollment date. Cervical exam results were defined by test type and the results (cytology: normal vs abnormal, HPV test: negative vs positive, and histology: high-grade abnormality vs normal and low-grade abnormality) and compared between the intervention and reference groups.

The cumulative incidence of having a cervical exam was estimated as 1 minus the survival curve from the Kaplan-Meier estimator. Similarly, the 95% CI was estimated as 1 minus the upper and lower limits of the CI of the survival curve. Differences between the survival curves were tested with the logrank test.

Hazard ratios (HRs) with 95% CIs were estimated with Cox proportional hazard models. As the proportionality assumption was not fulfilled in most of the models based on Schoenfeld residuals, we fitted separate models for the 0- to 6-month and 7- to 12-month windows. Statistical models were adjusted for education (no education or mandatory education only, high school diploma, or higher education [more than high school diploma]), yearly income (<US $23,271, US $23,272-US $40,726, US $40,727-US $58,181, US $58,182-US $81,467, or ≥US $81,468), country of birth (Norway, other Nordic countries, or other), employment status (employed, self-employed, or unemployed or outside of the workforce), and marital status (single, married or registered partner, or divorced, separated, or widowed), in addition to the matching variables of age and screening history at enrollment. Associations between exposure to the FightHPV app and a positive HPV test, abnormal cytology, and histologically confirmed high-grade abnormality were also adjusted for expected screening activity during the 1-year period after T0.

To study adherence to national screening recommendations, we classified the observed screening activity after T0 into three categories: (1) not due for a screening test (to study whether FightHPV app use was associated with overscreening), (2) due for a follow-up exam (to identify how well women with an abnormal screening result fulfilled the follow-up recommendation), or (3) due for a screening test.

To assess the possible influence of age on responding to the FightHPV app content, we stratified the study participants into three groups based on their age at the enrollment date (T0): (1) below screening age (participants aged <24 years), (2) younger screening age (participants aged 24 to 39 years), or (3) older screening age (participants aged >39 years).

### Ethics Approval

FightHPV is part of a project approved by the Norwegian Regional Ethics Committee (REK 2015/1926) and the institutional data protection officer. A liability and copyright terms and conditions of the game, along with logos from Oslo University Hospital and the Cancer Registry of Norway, have been incorporated into the app to enhance players’ trust in the content.

## Results

### Study Population Characteristics

Matching variables of age and screening history at enrollment were similar for both groups (Table 1). At enrollment, 89.75% (4055/4518) of the participants had had at least one cervical exam recorded. Of these 4055 participants, 2056 (50.7%) had only normal cytology or a negative HPV result, and 1999 (49.3%) had at least one abnormal cytology or a positive HPV test, histology result, or treatment. Compared with the women in the reference group (n=3860), those in the intervention group (n=658) were more likely to be in the higher-education category, that is, more than high school diploma (393/658, 59.7%, vs 1997/3860, 51.74%; *P*<.001); born in Norway (612/658, 93%, vs 3235/3860, 83.81%; *P*<.001); and single (348/658, 52.9%, vs 1687/3860, 43.7%; *P*<.001); in addition, the women in the intervention group were also more likely to have higher income (*P*=.002).

**Table 1:** Study population characteristics at enrollment by exposure status.

Variables						Intervention group (n=658)		Reference group (n=3860)		*P* value			
**Matching variables**													
	Age (years), median (IQR)				35 (28-45)		36 (28-45)		.63				
	**Screening history at enrollment, n (%)**										.13		
		Never screened			72 (10.9)		391 (10.1)						
		Only normal cytology or negative HPV^a^ test			289 (43.9)		1767 (45.8)						
		Abnormal cytology or positive HPV test but no histology ever			160 (24.3)		990 (25.6)						
		At least one histology result			77 (11.7)		463 (12)						
		Treatment for cervical abnormalities ever			60 (9.1)		249 (6.5)						
**Adjustment variables**													
	**Education, n (%)**												<.001
				No education or mandatory education only	79 (12)		649 (16.8)						
				Upper secondary	186 (28.3)		1214 (31.5)						
				Higher education	393 (59.7)		1997 (51.7)						
	**Yearly income (US $), n (%)**												.002
				<23,271	87 (13.2)		410 (10.6)						
				23,272 to 40,726	127 (19.3)		892 (23.1)						
				40,727 to 58,181	189 (28.7)		1179 (30.5)						
				58,182 to 81,467	166 (25.2)		1012 (26.2)						
				≥81,468	89 (13.5)		367 (9.5)						
	**Country of birth, n (%)**												<.001
			Norway		612 (93)		3235 (83.8)						
			Other Nordic countries		16 (2.4)		61 (1.6)						
			Other		30 (4.6)		564 (14.6)						
	**Employment status, n (%)**												.05
			Employed		530 (80.5)		2952 (76.5)						
			Self-employed		14 (2.1)		77 (2)						
			Unemployed or outside of the workforce		114 (17.3)		831 (21.5)						
	**Marital status, n (%)**												<.001
			Single		348 (52.9)		1687 (43.7)						
			Married or registered partner		229 (34.8)		1664 (43.1)						
			Divorced or separated or widowed		81 (12.3)		509 (13.2)						

^a^HPV: human papillomavirus.

### Impact of Exposure to the FightHPV App

**Figure 3:**
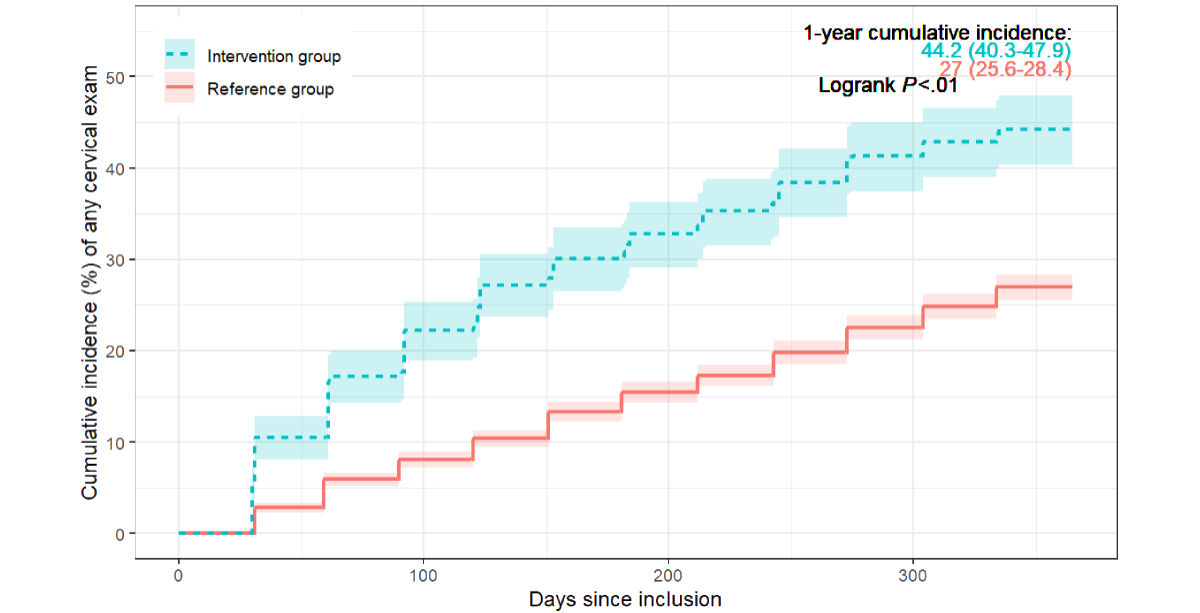
Cumulative incidence of having a cervical exam 1 year after enrollment among the intervention and reference groups with 95% CIs.

During the year after enrollment, 44.2% (95% CI 40.3%-47.9%) of the participants in the intervention group and 27% (95% CI 25.6%-28.4%) of the participants in the reference group had a cervical exam (*P*<.01; Figure 3). The results from the adjusted Cox model supported this observation, showing that exposure to the FightHPV app was associated with a higher number of cytology or HPV tests after the 0- to 6-month and 7- to 12-month periods after enrollment (HR 2.2, 95% CI 1.8-2.5 and HR 1.5, 95% CI 1.2-1.9, respectively; Table 2). The women in the intervention group were more likely to return for a diagnostic test than those in the reference group in both time periods (0-6 months: HR 3.8, 95% CI 2.4-6.2; 7-12 months: HR 3.6, 95% CI 1.9-6.7; Table 2).

**Table 2:** Association between exposure to the FightHPV app and having a cytology or HPV test, diagnostic test (histology), or any cervical exam 1 year after the enrollment date (T0) stratified by time since the enrollment date (T0). A woman could have had both screening and diagnostic exams during the same period.

Test and time period (months)		Intervention group (n=658), n (%)	Reference group (n=3860), n (%)	Unadjusted HR^a^ (95% CI)	Adjusted HR^b^ (95% CI)
**Cytology or HPV^c^ test**					
	0 to 6	195 (29.6)	585 (15.2)	2.2 (1.8-2.6)	2.2 (1.8-2.5)
	7 to 12	92 (19.9)	450 (13.7)	1.5 (1.2-1.9)	1.5 (1.2-1.9)
**Diagnostic test (histology)**					
	0 to 6	29 (4.4)	45 (1.2)	3.8 (2.4-6.1)	3.8 (2.4-6.2)
	7 to 12	16 (2.5)	31 (0.8)	3.2 (1.7-5.8)	3.6 (1.9-6.7)
**Any cervical** **exam** **(cytology,** **HPV** **test, or histology)**					
	0 to 6	208 (31.6)	596 (15.4)	2.3 (2.0-2.7)	2.3 (2.0-2.7)
	7 to 12	83 (18.4)	445 (13.6)	1.4 (1.1-1.8)	1.4 (1.1-1.8)

^a^HR: hazard ratio.

^b^Adjusted for education, country of birth, employment status, marital status, and income, in addition to the matching variables of screening history at enrollment and age.

^c^HPV: human papillomavirus.

**Figure 4:**
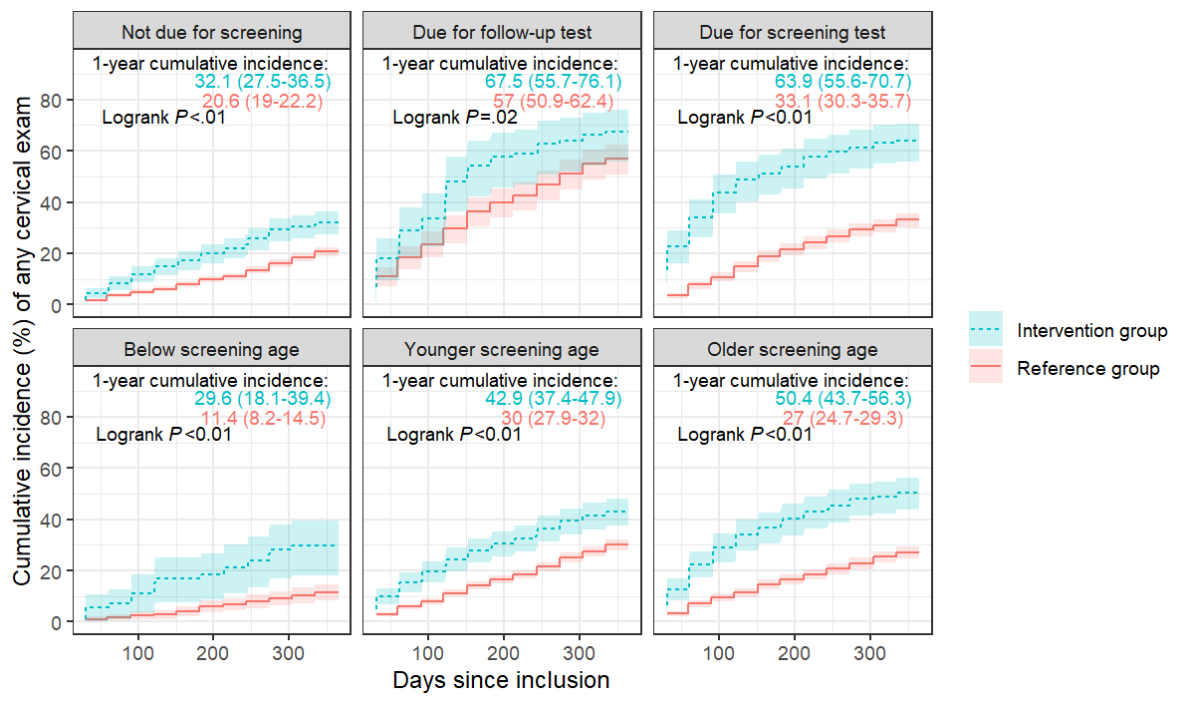
Cumulative incidence of having a cervical exam 1 year after enrollment among the intervention and reference groups with 95% CIs across expected screening activity subgroups and study participants’ age.

Exposure to the FightHPV app was associated with a higher number of cervical exams, independent of expected screening activities as determined by the national screening guidelines. Among the women who were due for a screening test, 63.9% (95% CI 55.6%-70.7%) in the intervention group and 33.1% (95% CI 30.3%-35.7%) in the reference group had a cervical exam 1 year after enrollment (*P*<.01; Figure 4). Exposure to the FightHPV app resulted in increased cervical exams during the first 6 months after enrollment (HR 3.3, 95% CI 2.6-4.3) but not during the 7- to 12-month period after enrollment (Table 3). Among the women who needed a follow-up exam, 67.5% (95% CI 55.7%-76.1%) in the intervention group and 57% (95% CI 50.9%-62.4%) in the reference group had a cervical exam during the 1-year period (*P*=.02; Figure 4). Exposure to the FightHPV app resulted in increased cervical exams during the first 6-month period after enrollment (HR 1.8, 95% CI 1.2-2.6) but not during the 7- to 12-month period after enrollment (Table 3). Among the women who were not due for a screening test, 32.1% (95% CI 27.5%-36.5%) in the intervention group and 20.6% (95% CI 19.5%-22.2%) in the reference group had a cervical exam in the 1-year period (*P*<.01; Figure 4), and exposure to the FightHPV app was associated with a higher number of cervical exams during both time periods (0-6 months: HR 1.9, 95% CI 1.5-2.5; 7-12 months: HR 1.5, 95% CI 1.1-2.0; Table 3).

**Table 3:** Association between exposure to the FightHPV app and having a cervical exam during the 1-year period after the enrollment date (T0) across expected screening activity subgroups, reflecting adherence to national screening recommendations, stratified by time since the enrollment date (T0).

			Any cervical exam (cytology, HPV^a^ test, or histology)		
			0 to 6 months		7 to 12 months
**Not due for screening test (n=2856)^b^**					
	Intervention group (n=416), n (%)	76 (18.3)		58 (17.1)	
	Reference group (n=2440), n (%)	239 (9.8)		264 (12)	
	Adjusted HR^c,d^ (95% CI)	1.9 (1.5-2.5)		1.5 (1.1-2.0)	
**Due for follow-up exam (n=367)^e^**					
	Intervention group (n=83), n (%)	47 (56.6)		9 (25)	
	Reference group (n=284), n (%)	113 (39.8)		49 (28.7)	
	Adjusted HR (95% CI)	1.8 (1.2-2.6)		1.1 (0.5-2.3)	
**Due for screening test (n=1295)^f^**					
	Intervention group (n=159), n (%)	85 (53.8)		16 (21.9)	
	Reference group (n=1136), n (%)	244 (21.5)		132 (14.8)	
	Adjusted HR (95% CI)	3.3 (2.6-4.3)		1.6 (0.9-2.7)	

^a^HPV: human papillomavirus.

^b^Not due for screening test: women aged <25 years; last cervical exam was <2.8 years before the enrollment date (T0); only normal test results during the last 3 years.

^c^HR: hazard ratio.

^d^Adjusted for education, country of birth, employment status, marital status, and income, in addition to the matching variables of screening history at enrollment and age.

^e^Due for follow-up exam: women at any age; had an abnormal primary cervical screening exam 3 years before the enrollment date (T0).

^f^Due for screening test: women aged >25 years; no cervical exam results <2.8 years before the enrollment date (T0).

The 1-year cumulative incidence of a cervical exam was significantly higher among those exposed to the FightHPV app, regardless of age. The proportion of women who had a cervical exam in the intervention group versus those in the reference group was 50.4% (95% CI 43.7%-56.3%) versus 27% (95% CI 24.7%-29.3%) in the older screening age group (*P*<.01), 42.9% (95% CI 37.4%-47.9%) versus 30% (95% CI 27.9%-32%) in the younger screening age group (*P*<.01), and 29.6% (95% CI 18.1%-39.4%) versus 11.4% (95% CI 8.2%-14.5%) among those below the screening age (*P*<.01), respectively (Figure 4). This difference was more pronounced during the first 6-month period after enrollment, being almost 3 times higher among those exposed to the FightHPV app in the older screening age group (HR 2.7, 95% CI 2.1-3.5), twice as high in the younger screening age group (HR 1.9, 95% CI 1.5-2.4), and 5 times higher among those below the screening age (HR 5.3, 95% CI 2.5-11.3; Table 4). In the 7- to 12-month period after enrollment, the magnitude of association for each age group was lower and only borderline significant for those below the screening age and the older screening age group (Table 4).

Comparing cervical exam outcomes, the women in the intervention group were 4 times more likely to test positive for HPV (HR 4.1, 95% CI 2.2-7.8), twice as likely to have abnormal cytology (HR 2.2, 95% CI 1.3-3.7), and 12 times more likely to be diagnosed with a high-grade abnormality (HR 12.7, 95% CI 5.0-32.5) compared with the women in the reference group during the first 6 months after enrollment (Table 5). During the 7- to 12-month period after enrollment, the women in the intervention group were 2 to 3 times more likely to test positive for HPV (HR 2.3, 95% CI 1.2-4.4), have abnormal cytology (HR 2.8, 95% CI 1.5-5.2), and be diagnosed with a high-grade abnormality (HR 3.0, 95% CI 1.0-9.0) compared with the women in the reference group (Table 5).

In addition, separate analyses on education, screening history, country of birth, and income were conducted (Multimedia Appendix 1), all of which confirmed a strong and consistent association between exposure to the FightHPV app and having a cervical exam within a year.

**Table 4:** Association between exposure to the FightHPV app and having a cervical exam during the 1-year period after the enrollment date (T0) across the study participants’ age and stratified by time since the enrollment date (T0).

		Any cervical exam (cytology, HPV^a^ test, or histology)	
		0 to 6 months	7 to 12 months
**Below screening age (n=465)^b^**			
	Intervention group (n=70), n (%)	13 (18.3)	8 (13.8)
	Reference group (n=395), n (%)	24 (6.1)	21 (5.7)
	Adjusted HR^c,d^ (95% CI)	5.3 (2.5-11.3)	2.4 (1.0-5.6)
**Younger screening age (n=2351)^e^**			
	Intervention group (n=344), n (%)	99 (28.9)	48 (19.7)
	Reference group (n=2007), n (%)	331 (16.5)	271 (16.2)
	Adjusted HR (95% CI)	1.9 (1.5-2.4)	1.2 (0.9-1.7)
**Older screening age (n=1702)^f^**			
	Intervention group (n=244), n (%)	96 (39.3)	27 (18.2)
	Reference group (n=1458), n (%)	241 (16.5)	153 (12.6)
	Adjusted HR (95% CI)	2.7 (2.1-3.5)	1.5 (1.0-2.3)

^a^HPV: human papillomavirus.

^b^Below screening age: women aged <24 years.

^c^HR: hazard ratio.

^d^Adjusted for education, country of birth, employment status, marital status, and income, in addition to the matching variables of screening history at enrollment and age.

^e^Younger screening age: women aged between 24 and 39 years.

^f^Older screening age: women aged >39 years.

**Table 5:** Association between exposure to the FightHPV app and having a positive human papillomavirus (HPV) test result, abnormal cytology, or high-grade abnormality diagnosis 1 year after the enrollment date (T0) stratified by time since the enrollment date (T0).

Test result and time period (months)			Intervention group (n=658), n (%)		Reference group (n=3860), n (%)		Unadjusted HR^a^ (95% CI)		Adjusted HR^b^ (95% CI)
**Positive** **HPV** **test**									
	0 to 6	20 (3)		23 (0.6)		5.2 (2.8-9.4)		4.1 (2.2-7.8)	
	7 to 12	15 (2.4)		31 (0.8)		2.9 (1.6-5.4)		2.3 (1.2-4.4)	
**Abnormal cytology**									
	0 to 6	24 (3.6)		48 (1.2)		3.0 (1.8-4.8)		2.2 (1.3-3.7)	
	7 to 12	16 (2.5)		33 (0.9)		2.9 (1.6-5.3)		2.8 (1.5-5.2)	
**High-grade abnormality**									
	0 to 6	16 (2.4)		6 (0.2)		15.8 (6.2-40.5)		12.7 (5.0-32.5)	
	7 to 12	5 (0.8)		12 (0.3)		2.5 (0.9-7.1)		3.0 (1.0-9.0)	

^a^HR: hazard ratio.

^b^Adjusted for education, country of birth, employment status, marital status, income and expected screening activity, in addition to the matching variables of screening history at enrollment and age.

## Discussion

### Principal Findings

To our knowledge, the FightHPV app is the first-ever game-based health-related–information intervention tool developed to increase demand for cancer screening among the screening program target population. The FightHPV app gamified scientifically accepted principles of cervical cancer prevention to augment the processing and contextualizing of health information, with the ultimate goal to nudge the player to attend screening. We demonstrated that women who used the FightHPV app were up to 3 times more likely to make an appointment and have a cervical exam than women who were not exposed to the app and its educational content, regardless of previous screening history or age.

Exposure to the FightHPV app had the highest impact on women during the first 6 months after exposure but continued to influence health-seeking behaviors, albeit to a lesser effect, during the 7- to 12-month period. This pattern was consistent across all expected screening activity and age categories. The desire to seek possibilities to have a cervical exam as soon as possible may reflect the unease regarding forgetting to book an appointment and, consequently, missing any potential cervical abnormalities that might need intervention. Studies have shown that exposure to information motivates positive health-seeking behaviors [7,28], highlighting the critical role of health literacy, in which awareness or knowledge of a health condition and its consequences can elicit certain behaviors among those affected by the condition. Health literacy is a concept emerging from social cognitive and social efficacy theories, which explain how perception and interpretation of health information influence behavior. Systematic reviews [29,30] and other studies [31] have highlighted that increased health literacy affects reproductive health behaviors and outcomes among women, especially those at risk for cancer.

We detected that the FightHPV app players had almost 13 times higher risk of being diagnosed with a histologically confirmed precancerous abnormality than the women in the reference group, whereas, in comparison, the overall attendance was improved approximately 3 times. This striking difference in participation in screening and the risk of those participating after exposure to the FightHPV app indicate that the game-based intervention influenced how users understood and interpreted the relevance and value of the screening test [32,33]. This is well in line with what we learned from focus group discussions during the development of the FightHPV app [19], where it was documented that the FightHPV app encouraged players to think about their behavior and reflect on the personal risks of cervical cancer. The observed higher rates of precancerous abnormalities in the intervention group suggests that those who perceived themselves to be at higher risk also attended the screening. This suggests that the FightHPV app not only increased awareness about the risks associated with cervical cancer but also helped to overcome emotional and practical barriers to attend the screening [22,32].

It is important to highlight that the reference and intervention cohorts were similar with regard to age and screening history—the latter is one of the strongest modifiers of cervical cancer risk. Furthermore, screening attendance is also used as a proxy to describe health consciousness and health behavior. Access to complete and accurate information on screening exams and the results for each participant allowed matching based on screening history at enrollment, which, in turn, ensured that both groups were comparable with respect to those never screened, those who had previously normal or abnormal exams, and those who had an invasive histology exam. Therefore, we argue that the observed differences cannot be explained by differences in cervical cancer risk and are rather a result of increased awareness from using the FightHPV app.

We also considered the possibility that the reported increase in the incidence of precancerous abnormalities over the last decades [34] might explain the higher rate of detection of high-grade abnormalities in the intervention group participants, who were enrolled in 2017, some 2 years after the reference group enrollment (2015). However, it is highly unlikely that birth cohorts 2 years apart represent differences in behavior of this magnitude. In addition, HPV-based screening with more aggressive clinical management algorithms had already been introduced in Norway in 2015 and therefore had a similar impact on the intervention and reference groups [35].

We have used cervical cancer screening as an example of a possible application field where knowledge translation via gamification and mobile phones can be used. In cervical cancer screening, women’s attitude toward screening, along with organizational factors, is crucial [36]. It has been found that the decision to skip the screening is usually not an active choice but rather a lack of knowledge or willingness to act [37]; hence, these interventions promoting these aspects should be targeted. Our results are in line with evidence that suggests that theory-based and culturally and linguistically sensitive educational interventions conducted by health advisors positively affect screening rates [18].

The application of game mechanics in this nongame context injected a little fun into health communications, as previously described [9,19]. In-game immediate feedback, in terms of cumulative scores and visible and auditable incentives after solving a puzzle or failing to do so, contributed to the game flow and allowed players to reach a state of absorption, which is believed to be an important aspect in the development of consciousness and enjoyment [4]. Solving puzzles provides an immediate feeling of achievement, and the player will gravitate toward more difficult puzzles. The great advantage of failing to solve a puzzle is that the player has an opportunity to repeat the puzzle and challenge. Short in-game messages in the FightHPV app provided narratives for each episode and at the end of each puzzle to increase contextualization of the information for the players.

The World Health Organization guideline (refer to the Introduction section) highlights the importance of taking advantage of digital technology interventions to contribute to health system improvements [1]. Among the 9 key recommendations, 1 is dedicated to targeted client communication for behavior change. According to the guideline, the transmission of health information via mobile phones is effective, acceptable, and uses fewer resources than nondigital interventions. This study adds to the growing body of evidence indicating that health-related information can be effectively distributed via mobile phones; however, it must be emphasized that interventions such as the FightHPV app will not serve the final goal of increasing cervical cancer screening attendance and hence reducing cervical cancer incidence and mortality without an existing and working screening program. In resource-constrained settings where access to screening is often nonexistent, the focus should first be on building up the screening program and improving health policy in general.

Our experience of developing the FightHPV app suggests that a successful team to customize the app would require a group of public health specialists, a graphic designer, and 1 or 2 mobile app developers. Together with a sociologist, the public health specialists can create the country- and language-specific content for in-game text messages, assess the game characters’ appropriateness, and, if needed, guide the graphic designer to make the adjustments. Once the content is confirmed, developers with Android and Swift programming experience can make the necessary modifications, and game testers in the target group can confirm app readiness. After the launch, a marketing strategy is required to get people interested in the app and ensure sustainable app uptake. Our experience showed that after a promotional video was published on the NCCSP Facebook page, there was a rapid increase in downloads, which subsided after a week (Figure S2 in Multimedia Appendix 1). The regular promotion of the app is crucial for a long-term effect on screening attendance.

### Strengths and Limitations

Our study was designed to leverage Norwegian high-quality registry-based data. Accurate information about outcomes and controlling variables obtained from the screening registry and Statistics Norway resulted in increased internal validity of the study. Although only 658 women living in Norway were enrolled to the intervention group, we believe this figure represents a subpopulation of women who are willing to change their screening behavior. We demonstrated that the intervention had a significant positive effect on the future screening–related decisions of this part of the population. We argue that this is the most crucial part of the population to reach because those unwilling to change their health behavior will not benefit from using this app. However, more studies are needed to replicate the observed effect of gamification on cancer screening in different health care settings and populations.

Despite the high number of total worldwide downloads, we only enrolled Norwegian citizens because it was crucial to obtain personal-level data from the Norwegian health registries, and it made the comparison of outcomes between the intervention and reference groups meaningful because both groups had to adhere to the same national cervical cancer screening guidelines (such as a 3-year screening interval and the starting age of screening), which typically differ across countries.

Because of the data protection law, we were not allowed to follow the participants’ progress in the game or played time. Therefore, we were not able to assess how the exposure time might have influenced future screening–related decisions. However, we assume that most of the people who confirmed their willingness to participate spent a reasonable time exploring the app and therefore were exposed to its educational content. Although we lacked data on censoring, such as emigration and death data, during the 1-year period after enrollment for the intervention group, we assume that this was indifferent and low among both the intervention and reference groups. If anything, the associations between exposure to the FightHPV app and screening participation might be somewhat underestimated, although sensitivity analyses indicated a minimal effect only.

### Conclusions

Exposure to the FightHPV app was significantly associated with the increased number of cervical exams across the various analyses. For the first time, we demonstrated the effectiveness of gamification combined with mobile technology in cancer prevention by empowering women to make an active health-related decision to attend cervical cancer screening. Gamification can significantly improve the understanding of complicated scientific concepts behind suggested interventions and increase the acceptance of proposed cancer control measures.

## Appendix

Multimedia Appendix 1 Additional information on study methods and results.

## Abbreviations

HPV: human papillomavirus
HR: hazard ratio
NCCSP: Norwegian Cervical Cancer Screening Program
