# Supplementary material for: Impact of the Mobile Game FightHPV on Cervical Cancer Screening Attendance: Retrospective Cohort Study
Source: JMIR Serious Games. 2022 Dec 13;10(4):e36197. doi: 10.2196/36197 (PMC9795393; doi:10.2196/36197)
Supplement: Multimedia Appendix 1 [file games_v10i4e36197_app1.docx]

**Multimedia Appendix 1**

**Description of the Norwegian Cervical Cancer Screening Program and the Linkage Process**

The Norwegian Cervical Cancer Screening Program (NCCSP) was established in 1995 at the Cancer Registry of Norway for evidence-based screening, diagnostics, and follow-up procedures. Since 1995, the NCCSP has recommended Norwegian female residents aged 25 to 69 years undergo cervical-cancer screening by cytology every three years. Along with the development of screening technology, the screening program has been modified, and since 2005, HPV testing has been used to follow-up mildly abnormal cytology’s (Figure S1). In 2019, NCCSP started a national rollout of HPV-based screening, recommending for women aged 34 to 69 years HPV testing every five years, whereas HPV-positive results were triaged with cytology. Women with abnormal cytology test were referred either for a follow-up exam in one year or an immediate diagnostic exam with colposcopic evaluation and biopsy. Those with histologically confirmed precancer or cancer are referred to an excisional procedure or cancer treatment, respectively.

NCCSP administers a nationwide data collection system, which includes close to 100% of all cervical cancer screening related clinical exams such as cytology and histology testing (pathology labs), HPV testing (microbiology and pathology labs), treatment of precancers (gynecology) and treatment of cancer (oncology). Data from both public and private health care sectors are included [24].

In Norway, cytology results were interpreted according to the Bethesda System [25] and histology results by Tavassoli et al [26]. For the present study, we categorized cytology results as normal (including only negative for intraepithelial lesions or malignancy) and abnormal (including cancer, high-grade squamous intraepithelial lesion (HSIL), atypical squamous cells cannot rule out HSIL, atypical glandular cells, low-grade squamous intraepithelial lesion, atypical squamous cells of undetermined significance). Histology results were categorized as high-grade (including cervical intraepithelial neoplasia grade 2 and 3, endocervical adenocarcinoma in situ, and cervical cancer) or normal. HPV tests results were categorized as negative or positive. Despite national recommendations, screening is at the discretion of the individual. As a result, the number of screening records and the time between screenings vary considerably between women, resulting in heterogenicity in terms of individual cervical cancer risk [27].

For each study participant, we used the unique, national identification number to combine information about individual cervical exams from NCCSP, and data about education, income, country of birth, employment, and marital status from Statistics Norway at the time of the start of the study. For data protection, national identification numbers were managed by authorized registry personnel only and were deidentified using study-specific allocation numbers.

**Description of the FightHPV App**

We have developed a mobile app FightHPV, a game-based learning tool that educates users about cervical cancer risk factors, infection with HPV, the risks associated with HPV infection, and existing preventive methods to avoid HPV infection and cervical cancer. The game technical description has been described in detail elsewhere. [19, 20] In short, the FightHPV app uses 14 animated characters with allocated game rules to gamify six selected topics relevant for understanding cervical cancer prevention and related health messages. Epithelial cells, presented as personalized adorable feminine characters, were placed on the board-puzzle at the start and the player's objective is to find a sequence of moves that brings the board to an end state where all original epithelial cells are placed alongside each other to create a tissue. This process is encountered with pitfalls such as infection by HPV and corresponding countermeasures. For instance, low-risk and high-risk HPV types are characters representing “the enemies” that destroy epithelial cells, while a condom, vaccine, antibody, screening, and conization characters are helpers who counteract HPV effects on epithelial cells. We created different start board states with increasing levels of difficulty and complexity of character interactions to create many pitfalls and treatment scenarios for the player hence educating players about the subtleties in HPV infection and cervical cancer screening and treatment at different stages of disease development. Based on the number of moves and time used, the player immediately received a score after completing the puzzle. To complete the mission and achieve the highest possible score, the player must apply game rules and use helpers to eliminate the virus. Gaming was bridged to the health context through short, integrated text messages during the game, explaining the epithelium’s protective purpose, and the devastating effect of HPV on the epithelial cells, the effect of vaccines by forming antibodies against HPV and how screening will help to repair the damaged epithelium. Different gameplay mechanics and strategies were employed in the game design to engage the player, learn the game rules, and practice useful thought patterns. It was important to provide a safe virtual environment to fail by not solving a puzzle within the given time, repeating the puzzle, applying helpers, and ultimately learning from failures. Players could see their scores on leaderboards, receive virtual awards such as batches, and present their achievements through social media. Before the app launch, the game was tested in focus groups and adjusted accordingly [19].

To increase visibility and attract more people to participate in the study, the FightHPV app launch was complimented with a short introductory film published on NCCSP Facebook page [21] that increased the number of downloads substantially (Figure S2). By the end of 2020, the film had received over 27,000 engagements and 1.9 million views. In addition, Cancer Registry of Norway and NCCSP promoted the app via their (social) media channels to increase perceived app reliability and credibility. During the first two years, the FightHPV app was downloaded around 20,000 times in 66 countries.


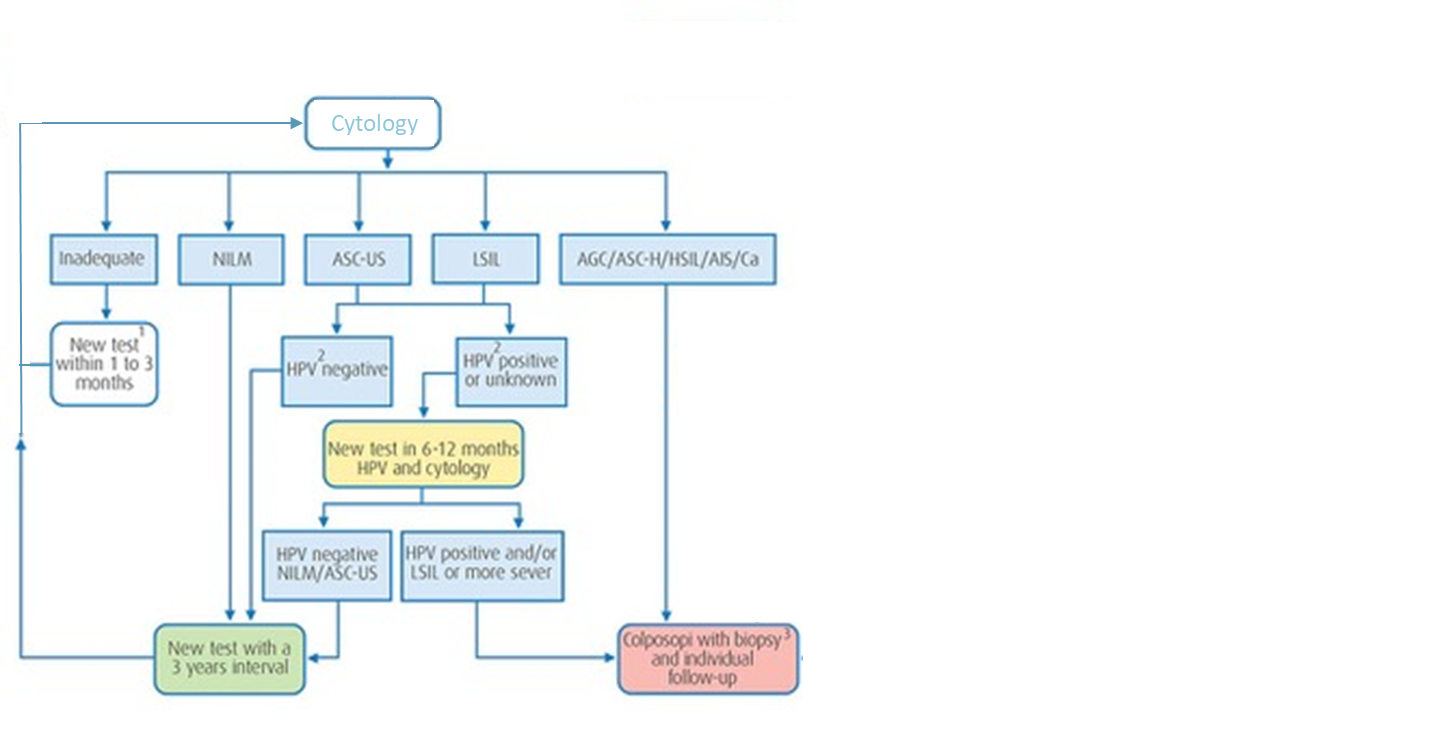


**Figure S1.** National cervical cancer screening recommendation during the study period. All women aged between 25 to 69-years old were invited triennially cytology-based screening. Women with inadequate cytology results at baseline were recommended to undergo another examination within 1-3 months, and those with cytology results of negative for intraepithelial lesions or malignancy (NILM) at baseline were referred to screening in 3 years. Women whose cytology results came back as high-grade cytological abnormalities (cancer, high-grade squamous intraepithelial lesion (HSIL), atypical squamous cells cannot rule out HSIL (ASC-H), atypical glandular cells (AGC) were referred to colposcopy. Women with cytology results of low-grade squamous intraepithelial lesion (LSIL) or atypical squamous cells of undetermined significance (ASC-US) had HPV testing performed on the residual cervical specimen. Those with negative HPV test results were referred to screening in 3 years; women with positive HPV test results were referred to increased surveillance (i.e., follow-up cytology and HPV testing within 6-12 months). Women who had follow-up cytology results of NILM or ASC-US and negative HPV test results were referred to screening in 3 years. Those with follow-up cytology results of LSIL or worse and/or positive HPV test results were referred to colposcopy.


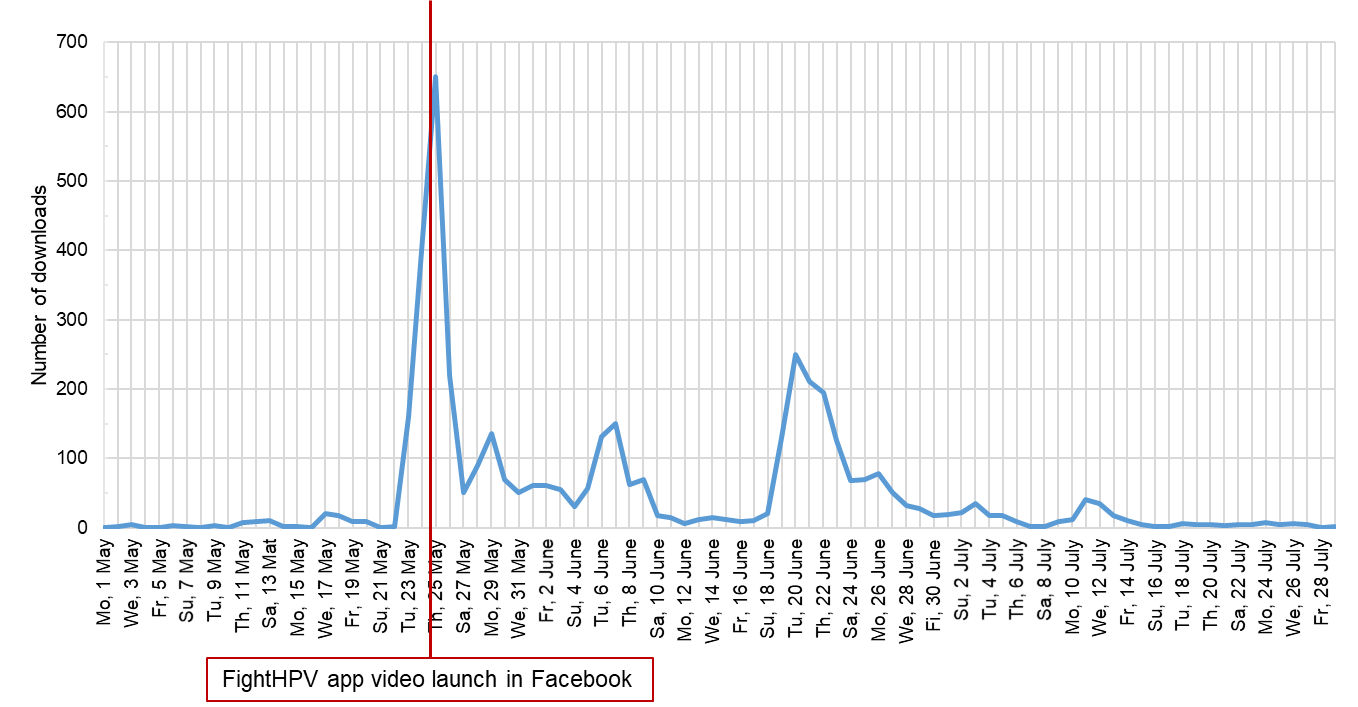


**Figure S2**. Number of total FightHPV app downloads in Norway from 1st of May until 30th of June 2017 with indication to Facebook promotion video launch.

**Table S1a.** Association between the FightHPV exposure and having a cervical exam during the one-year period after enrollment date (T_0_) stratified by study participants **education** and time since enrollment date (T_0_).

|  |  | **No education or mandatory**^a^ **only (N=728)** | | | **High school diploma**^a^ **(N=1400)** | | | **Higher education**^a^ **(N=2390)** | | |
| --- | --- | --- | --- | --- | --- | --- | --- | --- | --- | --- |
|  | Time period | Intervention group (n=79) | Reference group (n=649) | Adjusted HR^b^  95% CI | Intervention group (n=186) | Reference group (n=1214) | Adjusted HR^b^  95% CI | Intervention group (n=393) | Reference group (n=1997) | Adjusted HR^b^ 95% CI |
| Any cervical exam | 0–6 months | 21 (26.6%) | 83 (12.8%) | 2.4 (1.4-4.0) | 57 (30.6%) | 207 (17.1%) | 2.2 (1.6–3.0) | 130 (33.1%) | 306 (15.3%) | 2.4 (2.0–3.0) |
|  | 7–12 months | 10 (17.2%) | 60 (10.6%) | 1.9 (0.9-3.8) | 24 (18.6%) | 131 (13.0%) | 1.6 (1.0–2.5) | 49 (18.6%) | 254 (15.0%) | 1.3 (1.0–1.8) |

HR: hazard ratio, CI: confidence interval, any cervical exam: Cytology, HPV test or histology

^a^Mandatory education corresponds to completion of both primary and lower secondary education or the equivalent and having the right to three years’ full-time upper secondary education. High school diploma corresponds to completion of tertiary vocational education (both 2-years and 2-years or more), but which are not approved as higher education. Highereducation corresponds to completion of at least a tertiary education (more than high school diploma).

^b^Adjusted for country of birth, employment status, marital status, and income in addition to the matching variables

screening history and age.

**Table S1b.** Association between FightHPV app exposure and having a cervical exam during the one-year period after enrollment date (T_0_) stratified by screening activity subgroups, reflecting adherence to national screening recommendations, **education** and time since enrollment date (T_0_).

|  |  | **Not due for screening test (N=2856)** ^a^ | | | **Due for follow-up exam (N=367)** ^b^ | | | **Due for screening test (N=1295)^c^** | | |
| --- | --- | --- | --- | --- | --- | --- | --- | --- | --- | --- |
|  | Time period | Intervention group (n=416) | Reference group (n=2440) | Adjusted HR^d^  95% CI | Intervention group (n=83) | Reference group (n=284) | Adjusted HR^d^  95% CI | Intervention group (n=159) | Reference group (n=1136) | Adjusted HR^d^  95% CI |
| Not education or mandatory only^e^ (N=728) | 0–6 months | 8 (17.4%) | 38 (9.9%) | 2.3 (1.0–5.4) | 7 (53.8%) | 13 (30.2%) | 1.8 (0.6–5.6) | 6 (30.0%) | 32 (14.3%) | 3.0 (1.0–8.5) |
|  | 7–12 months | 6 (15.8%) | 38 (11.0%) | 1.6 (0.6–4.0) | 3 (50.0%) | 3 (10.0%) | NA | 1 (7.1%) | 19 (9.9%) | NA |
| High school diploma^e^ (N=1400) | 0–6 months | 12 (11.2%) | 80 (11.0%) | 1.1 (0.6–2.1) | 15 (55.6%) | 36 (41.4%) | 2.0 (0.9–4.2) | 30 (57.7%) | 91 (22.6%) | 3.7 (2.4–5.7) |
|  | 7–12 months | 13 (13.7%) | 79 (12.2%) | 1.3 (0.7–2.3) | 5 (41.7%) | 18 (35.3%) | 2.0 (0.4–10.2) | 6 (27.3%) | 34 (10.9%) | 2.6 (1.1–6.5) |
| Higher education^e^ (N = 2390) | 0–6 months | 56 (21.2%) | 121 (9.1%) | 2.4 (1.8–3.4) | 25 (58.1%) | 64 (41.6%) | 1.8 (1.1–3.0) | 49 (57.0%) | 121 (23.7%) | 3.3 (2.4–4.7) |
|  | 7–12 months | 39 (18.8%) | 147 (12.1%) | 1.7 (1.2–2.4) | 1 (5.6%) | 28 (31.1%) | NA | 9 (24.3%) | 79 (20.3%) | 1.4 (0.7–2.9) |

HR: hazard ratio, CI: confidence interval, any cervical exam: Cytology, HPV test or histology

^a^Not due for screening test – women under 25 years of age; last given cervical exam was less than 2.8 years before prior enrollment date (T_0_); only normal test results during the last 3 years.

^b^Due for a follow-up exam – women at any age; had an abnormal primary cervical screening exam 3 years prior enrollment date (T_0_).

**^c^**Due for screening test – women over 25 years of age; no cervical exam results 2.8 years prior enrollment date (T_0_).

^d^Adjusted for education, country of birth, employment status, marital status, and income in addition to the matching variables of screening history at enrollment and age.

^e^Mandatory education corresponds to completion of both primary and lower secondary education or the equivalent and having the right to three years’ full-time upper secondary education. High school diploma corresponds to completion of tertiary vocational education (both 2-years and 2-years or more), but which are not approved as higher education. Higher education corresponds to completion of at least a tertiary education (more than high school diploma).

**Table S1c.** Association between FightHPV app exposure and having any cervical exam during the one-year period after enrollment date (T_0_) stratified by participants age, **education,** and time since enrollment date (T_0_).

|  |  | **Under screening age (N=465)** ^a^ | | | **Younger screening age (N=2351)^b^** | | | **Older screening age (N=1702)^c^** | | |
| --- | --- | --- | --- | --- | --- | --- | --- | --- | --- | --- |
|  | Time period | Intervention group (n=70) | Reference group (n=395) | Adjusted HR^d^  95% CI | Intervention group (n=344) | Reference group (n=2007) | Adjusted HR^d^  95% CI | Intervention group (n=244) | Reference group (n=1458) | Adjusted HR^d^  95% CI |
| No education or mandatory only^e^ (N=728) | 0–6 months | 1 (12.5%) | 5 (6.5%) | NA | 13 (27.7%) | 39 (13.5%) | 2.3 (1.1–4.8) | 7 (29.2%) | 39 (13.7%) | 3.3 (1.3–8.5) |
|  | 7–12 months | 0 (0%) | 5 (6.9%) | NA | 8 (23.5%) | 30 (12.0%) | 2.8 (1.2–6.8) | 2 (11.8%) | 25 (10.2%) | 1.1 (0.2–5.1) |
| High school diploma^e^ (N=1400) | 0–6 months | 4 (14.8%) | 5 (4.7%) | 4.2 (0.7–25.6) | 22 (27.5%) | 85 (16.0%) | 2.1 (1.3–3.4) | 31 (39.2%) | 117 (20.3%) | 2.3 (1.6–3.5) |
|  | 7–12 months | 5 (21.7%) | 6 (5.9%) | 3.3 (0.9–12.8) | 7 (12.1%) | 68 (15.2%) | 0.8 (0.4–1.8) | 12 (25.0%) | 57 (12.4%) | 2.6 (1.4–5.0) |
| Higher education^e^  (N=2390) | 0–6 months | 8 (22.2%) | 14 (6.6%) | 5.8 (2.2–15.8) | 64 (29.6%) | 207 (17.4%) | 1.8 (1.4–2.4) | 58 (41.1%) | 85 (14.2%) | 2.6 (1.4–5.0) |
|  | 7–12 months | 3 (10.7%) | 10 (5.1%) | 1.5 (0.4–6.2) | 33 (21.7%) | 173 (17.6%) | 1.2 (0.9–1.9) | 13 (15.7%) | 71 (13.9%) | 1.1 (0.6–2.1) |

HR: hazard ratio, CI: confidence interval, any cervical exam: Cytology, HPV test or histology

^a^ Under screening age – women under 24 years of age.

**^b^**Younger screening age – women between 24 and 39 years of age.

**^c^**Older screening age – women over 39 years of age.

^d^Adjusted for education, country of birth, employment status, marital status, and income in addition to the matching variables of screening history at enrollment and age.

^e^Mandatory education corresponds to completion of both primary and lower secondary education or the equivalent and having the right to three years’ full-time upper secondary education. High school diploma corresponds to completion of tertiary vocational education (both 2-years and 2-years or more), but which are not approved as higher education. Higher education corresponds to completion of at least a tertiary education (more than high school diploma).

**Table S2a.** Association between the FightHPV exposure and having a cervical exam during the one-year period after enrollment date (T_0_) stratified by study participants **country of birth,** and time since enrollment date (T_0_).

|  |  | **Born in Norway (N=3847)** | | | **Born outside of Norway (N=671)** | | |
| --- | --- | --- | --- | --- | --- | --- | --- |
|  | Time period | Intervention group (n=612) | Reference group (n=3235) | Adjusted HR^a^  95% CI | Intervention group (n=46) | Reference group (n=625) | Adjusted HR^a^  95% CI |
| Any cervical exam | 0–6 months | 196 (32.0%) | 512 (15.8%) | 2.4 (2.0-2.8) | 12 (26.1%) | 84 (13.4%) | 2.3 (1.2–4.4) |
|  | 7–12 months | 79 (19.0%) | 388 (14.2%) | 1.4 (1.1-1.8) | 4 (11.8%) | 57 (10.5%) | 1.1 (0.4–3.2) |

HR: hazard ratio, CI: confidence interval, any cervical exam: Cytology, HPV test or histology

^a^Adjusted for country of birth, employment status, marital status, and income in addition to the matching variables

screening history and age.

**Table S2b.** Association between FightHPV app exposure and having a cervical exam during the one-year period after enrollment date (T_0_) stratified by screening activity subgroups, reflecting adherence to national screening recommendations, **country of birth,** and time since enrollment date (T_0_).

|  |  | **Not due for screening test (N=2856)^a^** | | | **Due for follow-up exam (N=367)^b^** | | | **Due for screening test (N=1295)^c^** | | |
| --- | --- | --- | --- | --- | --- | --- | --- | --- | --- | --- |
|  | Time period | Intervention group (n=416) | Reference group (n=2440) | Adjusted HR^d^  95% CI | Intervention group (n=83) | Reference group (n=284) | Adjusted HR^d^  95% CI | Intervention group (n=159) | Reference group (n=1136) | Adjusted HR^d^  95% CI |
| Born in Norway (N=3847) | 0–6 months | 71 (18.4%) | 205 (9.9%) | 2.0 (1.6–2.7) | 45 (57.0%) | 96 (39.5%) | 1.8 (1.2–2.5) | 80 (54.4%) | 211 (23.0%) | 3.3 (2.6–4.3) |
|  | 7–12 months | 55 (17.5%) | 233 (12.5%) | 1.5 (1.1–2.1) | 8 (23.5%) | 41 (27.9%) | 1.1 (0.5–2.5) | 16 (23.5%) | 114 (16.1%) | 1.7 (1.0–2.9) |
| Born outside of Norway (N=671) | 0–6 months | 5 (15.6%) | 34 (9.3%) | 1.7 (0.6–5.1) | 2 (50.0%) | 17 (41.5%) | 1.8 (0.3–11.7) | 5 (50.0%) | 33 (15.0%) | 5.3 (1.7–16.7) |
|  | 7–12 months | 3 (11.1%) | 31 (9.4%) | 1.4 (0.4–5.1) | 1 (50.0%) | 8 (33.3%) | NA | 0 (0%) | 18 (9.6%) | NA |

HR: hazard ratio, CI: confidence interval, any cervical exam: Cytology, HPV test or histology

^a^Not due for screening test – women under 25 years of age; last given cervical exam was less than 2.8 years before prior enrollment date (T_0_); only normal test results during the last 3 years.

**^b^**Due for a follow-up exam – women at any age; had an abnormal primary cervical screening exam 3 years prior enrollment date (T_0_).

**^c^**Due for screening test – women over 25 years of age; no cervical exam results 2.8 years prior enrollment date (T_0_).

^d^Adjusted for education, country of birth, employment status, marital status, and income in addition to the matching variables of screening history at enrollment and age.

**Table S2c.** Association between FightHPV app exposure and having any cervical exam during the one-year period after enrollment date (T_0_) stratified by participants age, **country of birth,** and time since enrollment date (T_0_).

|  |  | **Under screening age (N=465)^a^** | | | **Younger screening age (N=2351)^b^** | | | **Older screening age (N=1702)^c^** | | |
| --- | --- | --- | --- | --- | --- | --- | --- | --- | --- | --- |
|  | Time period | Intervention group (n=70) | Reference group (n=395) | Adjusted HR^d^  95% CI | Intervention group (n=344) | Reference group (n=2007) | Adjusted HR^d^  95% CI | Intervention group (n=244) | Reference group (n=1458) | Adjusted HR^d^  95% CI |
| Norwegian (N=3847) | 0–6 months | 13 (19.1%) | 19 (5.6%) | 6.1 (2.8–13.4) | 96 (29.9%) | 279 (17.1%) | 2.0 (1.6–2.5) | 87 (61.0%) | 214 (16.9%) | 2.7 (2.1–3.5) |
|  | 7–12 months | 8 (14.5%) | 20 (6.2%) | 2.4 (1.0–5.6) | 44 (19.6%) | 231 (17.1%) | 1.2 (0.9–1.7) | 27 (19.9%) | 137 (13.0%) | 1.7 (1.1–2.5) |
| Non-Norwegian (N=671) | 0–6 months | 0 (0%) | 5 (9.6%) |  | 3 (13.6%) | 52 (13.7%) | 1.4 (0.4–4.8) | 9 (42.9%) | 27(13.9%) | 3.8 (1.5–10.0) |
|  | 7–12 months | 0 (0%) | 1 (2.1%) |  | 4 (21.1%) | 40 (12.2%) | 1.9 (0.6–5.7) | 0 (0%) | 16 (9.6%) |  |

HR: hazard ratio, CI: confidence interval, any cervical exam: Cytology, HPV test or histology

^a^Under screening age – women under 24 years of age.

**^b^**Younger screening age – women between 24 and 39 years of age.

**^c^**Older screening age – women over 39 years of age.

^d^Adjusted for education, country of birth, employment status, marital status, and income in addition to the matching variables of screening history at enrollment and age.

**Table S3a.** Association between the FightHPV exposure and having a cervical exam during the one-year period after enrollment date (T_0_) stratified by study participants **income,** and time since enrollment date (T_0_).

|  |  | **Income < $40,726 (N=1516)** | | | **Income $40,727 – $58,181 (N=1368)** | | | **Income > $58,182 (N=1634)** | | |
| --- | --- | --- | --- | --- | --- | --- | --- | --- | --- | --- |
|  | Time period | Intervention group (n=214) | Reference group (n=1302) | Adjusted HR^a^  95% CI | Intervention group (n=189) | Reference group (n=1179) | Adjusted HR^a^  95% CI | Intervention group (n=255) | Reference group (n=1379) | Adjusted HR^a^  95% CI |
| Any cervical exam | 0-6 months | 43 (20.1%) | 154 (11.8%) | 1.8 (1.3-2.6) | 58 (30.7%) | 193 (16.4%) | 2.2 (1.6–2.9) | 107 (42.0%) | 249 (18.1%) | 2.9 (2.3–3.6) |
|  | 7-12 months | 28 (16.4%) | 122 (10.6%) | 1.6 (1.1-2.5) | 31 (23.7%) | 144 (14.6%) | 1.7 (1.1–2.5) | 24 (16.2%) | 179 (15.8%) | 1.0 (0.8–1.6) |

HR: hazard ratio, CI: confidence interval, any cervical exam: Cytology, HPV test or histology

^a^Adjusted for country of birth, employment status, marital status, and income in addition to the matching variables

screening history and age.

**Table S3b.** Association between FightHPV app exposure and having a cervical exam during the one-year period after enrollment date (T_0_) stratified by screening activity subgroups, reflecting adherence to national screening recommendations, **income,** and time since enrollment date (T_0_).

|  |  | **Not due for screening test (N=2856)**^a^ | | | **Due for follow-up exam (N=367) ^b^** | | | **Due for screening test (N=1295)^c^** | | |
| --- | --- | --- | --- | --- | --- | --- | --- | --- | --- | --- |
|  | Time period | Intervention group (n=416) | Reference group (n=2440) | Adjusted HR^d^  95% CI | Intervention group (n=83) | Reference group (n=284) | Adjusted HR^d^  95% CI | Intervention group (n=159) | Reference group (n=1136) | Adjusted HR^d^  95% CI |
| Income < $40,726 (N=1516) | 0–6 months | 20 (13.4%) | 61 (7.3%) | 2.1 (1.2–3.5) | 12 (42.9%) | 34 (36.6%) | 1.0 (0.5–2.0) | 11 (29.7%) | 59 (15.6%) | 2.2 (1.1–4.4) |
|  | 7–12 months | 21 (16.3%) | 77 (10.0%) | 1.8 (1.1–2.9) | 4 (25.0%) | 12 (20.3%) | 1.4 (0.4–5.1) | 3 (11.5%) | 33 (10.3%) | 0.9 (0.3–3.2) |
| Income $40,727 – $58,181 (N=1368) | 0–6 months | 18 (15.8%) | 74 (10.2%) | 1.6 (1.0–2.8) | 14 (60.9%) | 37 (42.5%) | 1.7 (0.8–3.3) | 26 (50.0%) | 82 (22.5%) | 2.8 (1.7–4.5) |
|  | 7–12 months | 17 (17.7%) | 86 (13.1%) | 1.4 (0.8–2.3) | 5 (55.6%) | 15 (30.0%) | 2.0 (0.6–7.1) | 9 (34.6%) | 43 (15.2%) | 2.6 (1.2–5.4) |
| Income > $58,182 (N=1634) | 0–6 months | 38 (24.7%) | 104 (11.8%) | 2.2 (1.5–3.2) | 21 (65.6%) | 42 (40.4%) | 2.5 (1.4–4.5) | 48 (69.6%) | 103 (26.1%) | 4.2 (2.9–6.0) |
|  | 7–12 months | 20 (17.2%) | 101 (13.0%) | 1.4 (0.8–2.2) | 0 (0%) | 22 (35.5%) | NA | 4 (19.0%) | 56 (19.2%) | 1.2 (0.4–3.3) |

HR: hazard ratio, CI: confidence interval, any cervical exam: Cytology, HPV test or histology

^a^Not due for screening test – women under 25 years of age; last given cervical exam was less than 2.8 years before prior enrollment date (T_0_); only normal test results during the last 3 years.

**^b^**Due for a follow-up exam – women at any age; had an abnormal primary cervical screening exam 3 years prior enrollment date (T_0_).

**^c^**Due for screening test – women over 25 years of age; no cervical exam results 2.8 years prior enrollment date (T_0_).

^d^Adjusted for education, country of birth, employment status, marital status, and income in addition to the matching variables of screening history at enrollment and age.

**Table S3c.** Association between FightHPV app exposure and having any cervical exam during the one-year period after enrollment date (T_0_) stratified by participants age, **income,** and time since enrollment date (T_0_).

|  |  | **Under screening age (N=465)** ^a^ | | | **Younger screening age (N=2351) ^b^** | | | **Older screening age (N=1702)^c^** | | |
| --- | --- | --- | --- | --- | --- | --- | --- | --- | --- | --- |
|  | Time period | Intervention group (n=70) | Reference group (n=395) | Adjusted HR^d^  95% CI | Intervention group (n=344) | Reference group (n=2007) | Adjusted HR^d^  95% CI | Intervention group (n=244) | Reference group (n=1458) | Adjusted HR^d^  95% CI |
| Income < $40,726 (N=1516) | 0–6 months | 11 (17.7%) | 20 (7.0%) | 4.8 (2.2–10.8) | 23 (20.4%) | 81 (13.4%) | 1.4 (0.8–2.2) | 9 (23.1%) | 53 (12.8%) | 2.1 (1.1–4.4) |
|  | 7–12 months | 8 (15.7%) | 17 (6.4%) | 2.5 (1.0–6.0) | 15 (16.7%) | 66 (12.6%) | 1.3 (0.7–2.3) | 5 (16.7%) | 39 (10.8%) | 1.9 (0.7–4.9) |
| Income $40,727 – $58,181 (N=1368) | 0–6 months | 1 (14.3%) | 4 (4.7%) | NA | 29 (25.9%) | 115 (16.9%) | 1.8 (1.2–2.7) | 28 (40.0%) | 74 (17.9%) | 2.7 (1.7–4.2) |
|  | 7–12 months | 0 (0%) | 3 (3.7%) | NA | 22 (26.5%) | 102 (18.1%) | 1.5 (0.9–2.3) | 9 (21.4%) | 39 (11.5%) | 2.0 (0.9–4.1) |
| Income > $58,182 (N=1634) | 0–6 months | 1 (50.0%) | 0 (0%) | NA | 47 (39.8%) | 135 (18.6%) | 2.6 (1.8–3.6) | 59 (43.7%) | 114 (18.1%) | 3.1 (2.3–4.3) |
|  | 7–12 months | 0 (0%) | 1 (4.5%) | NA | 11 (15.5%) | 103 (17.4%) | 0.8 (0.5–1.6) | 13 (17.1%) | 75 (14.5%) | 1.3 (0.7–2.3) |

HR: hazard ratio, CI: confidence interval, any cervical exam: Cytology, HPV test or histology

^a^Under screening age – women under 24 years of age.

**^b^**Younger screening age – women between 24 and 39 years of age.

**^c^**Older screening age – women over 39 years of age.

^d^Adjusted for education, country of birth, employment status, marital status, and income in addition to the matching variables of screening history at enrollment and age.
